# Combination of extracts from *Aristolochia cymbifera* with streptomycin as a potential antibacterial drug

**DOI:** 10.1186/2193-1801-2-430

**Published:** 2013-09-03

**Authors:** Willer F Silva, Samyra G Cecílio, Cintia LB Magalhães, Jaqueline MS Ferreira, Antonio H Tótola, Jose C de Magalhaes

**Affiliations:** Programa de Educação Tutorial (PET-DPCFC), Universidade Federal de São João Del-Rei, Campus Alto Paraopeba, Ouro Branco, Minas Gerais Brazil; Programa de Pós-graduação em Tecnologias para o desenvolvimento sustentável (PGTDS), Universidade Federal de São João Del-Rei, Campus Alto Paraopeba, Ouro Branco, Minas Gerais Brazil; Departamento de Ciências Biológicas (DECBI), Universidade Federal de Ouro Preto, Ouro Preto, Minas Gerais Brazil; Campus Centro Oeste Dona Lindu, Universidade Federal de São João del-Rei, Divinópolis, Minas Gerais Brazil; Departamento de Química, Biotecnologia e Engenharia de Bioprocessos (DQBIO), Universidade Federal de São João Del-Rei, Campus Alto Paraopeba, Rodovia MG 443, Km 07, Congonhas/Ouro Branco, Ouro Branco, Minas Gerais CEP 36420-000 Brazil

**Keywords:** *Aristolochia cymbifera*, Antibacterial activity, Synergism, Cytotoxicity, Drug discovery, Natural products

## Abstract

The appearance of new antibiotic-resistant bacteria is a societal problem that requires the development of new alternative treatments. Therefore, this work evaluated the antibacterial activity of ethanolic (EHI), dichloromethanic (EDI) and hexanic (EHE) extracts from *Aristolochia cymbifera* stems and the combination of these extracts with an antimicrobial drug to develop a new antibacterial therapy. The EDI, EHE and EHI extracts were obtained by maceration using three different solvents. The minimal inhibitory concentrations (MIC) of these extracts were determined using the microdilution test to determine the antibacterial potential of these extracts and their combination with streptomycin against *Staphylococcus aureus*, *Bacillus cereus*, *Klebsiella pneumoniae* and *Shigella flexneri*. The extract dose leading to the cytotoxicity of 50% of the cells (CC50) was evaluated using mammalian cells MA104 and the 3-(4,5-dimethylthiazol-2-yl)-2,5-diphenyltetrazolium bromide (MTT) colorimetric assay. The extracts had a MIC under 500 mg/L and a CC50 lower than 50 mg/L. The antibiotic/extract proportion influenced the antibacterial activity of the mixtures, and the proportion that optimized the antibacterial activity of streptomycin was a mixture that contained 75 percent of extract. This composition included less than 6.5 mg/L of extract and 2.5 mg/L of streptomycin and has potential as a new antibacterial therapy.

**Electronic supplementary material:**

The online version of this article (doi:10.1186/2193-1801-2-430) contains supplementary material, which is available to authorized users.

## Background

Longstanding experiments and the popular use of plants as therapeutic tools have helped to introduce chemical entities in modern medicine. Plants, especially those with ethnopharmacological uses, have been the main sources for the discovery of new drugs. Worldwide, natural products have played a prominent role in the prevention and treatment of several diseases for thousands of years (Chin *et al.*^2006^).

Drugs based on natural products have been developed from sources that include terrestrial plants, microorganisms, marine organisms, terrestrial vertebrates and invertebrates (Newman *et al.*^2000^). Brazil has a vast forest and a popular tradition associated with the use of medicinal plants for antimicrobial applications (Sartoratto *et al*. 2004). Thus, extracts from plants can be used as sources of new drugs or antimicrobial compounds (Alviano and Alviano 2009) and these new therapies are of great importance, as the emergence of resistant strains has increased the difficulty of treating infections (Cole *et al*. 2003).

*Staphylococcus aureus* (*S. aureus*), *Bacillus cereus* (*B. cereus*), *Klebsiella pneumoniae* (*K. pneumoniae*) and *Shigella flexneri* (*S. flexneri*) are known to cause enteric disease. *Shigella flexneri* causes enteric shigellosis and is responsible for 1.1 million deaths per year, with children being the most affected group (Jennison and Verma 2004). *B. cereus* is responsible for diarrheal food poisoning due to its toxin production (Ceuppens *et al.*^2012^). *Staphylococcus aureus* can induce many diseases, such as bovine mastitis, skin disease and gastroenteritis, due to the ingestion of enterotoxins (Reinhardt *et al.*^2013^; Moran *et al.*^2013^; Hyeon *et al.*^2013^) and are often associated with antibiotic resistance. Another species that has been shown to be antibiotic resistant is *Klebsiella pneumoniae,* including carbapenem-resistant *Klebsiella pneumoniae* (CRKP) (Tenover 2006), which can inactivate the carbapenems.

In some regions, enteric diseases are treated with teas, infusions or macerated medicinal plants and this folk medical knowledge is transmitted from generation to generation. This alternative treatment, which sometimes represents the only therapy option for many people, is widely used due to its low cost and easy access (Veiga et al. 2005).

Species of the *Aristolochia* genus are used in traditional medicine, mainly in South America, to treat skin diseases, poisoning, wounds, worms, diarrhea, and also as emmenagogues. The species *Aristolochia cymbifera* (*Aristolochiaceae*) is popularly known as jarrinha, papo de peru, cipó mil homens, cassaú and other common names (Wu *et al.*^2004^). Some studies using *A. cymbifera* extracts have indicated potential antimicrobial properties of this species. In experiments performed by Carvalho *et al*. (2008), the leaf methanol extract of this species showed strong activity against promastigotes from *Leishmania chagasi,* and the diterpene copalic acid was isolated using bioassay-guided fractionation and showed high toxicity against the extracellular form of the parasite. Sartorelli *et al*. (2010) observed that copalic acid was also active against trypomastigotes of *Trypanosoma cruzi*. Copalic acid maintained its selectivity against *T. cruzi,* although it showed low toxicity against infected mammalian cells.

In studies conducted by Alviano *et al*. (2008) that evaluated the antioxidant potential and antimicrobial properties of plants used in Brazilian folk medicine, the crude ethanol-water (1:1) extract from *A. cymbifera* stems exhibited a significant antibacterial effect. This extract inhibited the growth of the oral bacteria *Prevotella intermedia*, *Porphyromonas gingivalis*, *Fusobacterium nucleatum*, *Lactobacillus casei* and *Streptococcus mutans* even in the presence of an artificial biofilm. In experiments using the agar dilution method, the ethanolic extract from *A. cymbifera* stems inhibited the growth of various staphylococci, including multi-drug resistant endemic Brazilian clones of *Staphylococcus aureus*. The hexane fraction of the extract proved to be active against *Pseudomonas aeruginosa* strains Machado *et al*. 2005).

Thus, based on the potential and promising use of medicinal plants in traditional medicine, the current investigation involved screening the ethanolic (EHI), dichloromethanic (EDI) and hexanic (EHE) extracts of *A. cymbifera* against important pathogenic bacteria and combining these extracts with streptomycin determine the prospects for a new antibacterial therapy.

## Results and discussion

### Results

#### Extract absorption outlines

**Figure 1:**
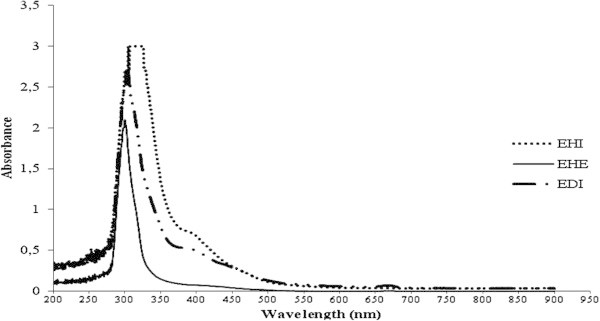
**UV/Vis absorption spectra (200 to 900 nm) of EDI, EHE and EHI.** EHE showed the smallest absorption band at 316 ± 6 nm. EDI and EHI had similar profiles and showed an absorption band at 400 nm that was not observed in the EHE spectrum.

The three extracts from *A. cymbifera* had differences in their absorption spectra, mainly in the UV region (Figure 1). In the EHE and EDI extracts, the most intense absorption bands were observed at 300 ± 2 and 303 ± 3 nm, respectively. The absorbance of EHE at these wavelengths was lower than that of EDI. An absorption band at 400 nm was also observed in EHI and EDI.

#### Determination of Minimum Inhibitory Concentration (MIC)

The MIC values found for the three extracts are presented in Table 1. Values less than or equal to 500 mg L^-1^ were considered to be the MIC. A MIC of less than or equal to 100 mg L^-1^ was considered as potential for prospect a new therapy (Rios and Recio 2005). *B. cereus* appeared to be the most sensitive bacteria to these three extracts, with a MIC lower than 100 mg L^-1^. The hydrophilic extracts (EDI and EHI) were more active against *S. aureus* and *S. flexneri* than was the hydrophobic hexanic extract (EHE).

**Table 1:** **Minimum inhibitory concentration (mg L**^**-1**^**) of ethanolic (EHE), dichloromethanic (EDI) and hexanic (EHE) extracts from*Aristolochia cymbifera***

Bacteria	Extracts from ***Aristolochia cymbifera***					
	EDI		EHE		EHI	
	X*	SD †	X*	SD †	X*	SD †
*S. aureus*	214.29	60.99	>500	>500	160.71	60.99
*B. cereus*	89.29	33.41	62.50	0.00	62.50	0.00
*K. pneumoniae*	416.67	129.10	250.00	0.00	500.00	0.00
*S. flexneri*	166.67	64.55	395.83	166.15	200.00	68.47

*X, Average MIC values obtained from replicates.

† SD, standard deviation associated with the average obtained from the replicates.

#### Effect of combination of streptomycin and A. cymbifera extracts

Different effects were observed when the streptomycin/extract proportions were changed, and an increase in the antibacterial activity was observed when the percentage of extracts in the mixture was higher (see Additional file 1). A mixture containing 75% extract reduced the MIC value of streptomycin approximately four-fold against *S. aureus*, *K. pneumoniae*, *B. cereus* and *S. flexneri*.

**Figure 2:**
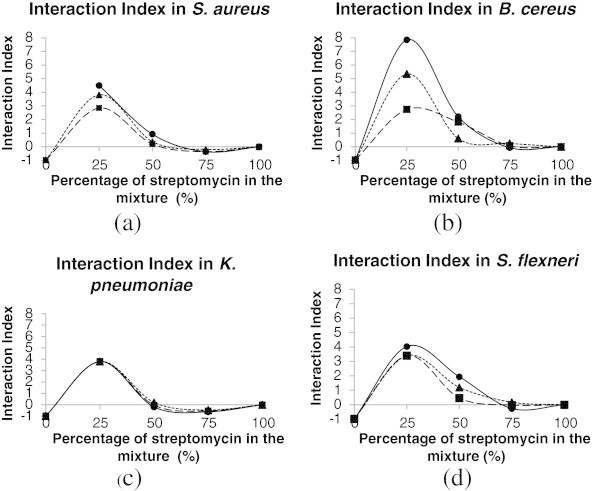
**Interaction indexes (II) of the EDI (■), EHE (●) and EHI (▲) extracts as a function of the percentage of streptomycin in the mixture for*S. aureus*(a),*B. cereus*(b),*K. pneumoniae*(c) and*S. flexneri*(d).** The optimum proportion of the mixture was 75% extract (II maximum). There was a large difference in the II of EHE between the *B. cereus* and the other bacteria. There was no difference in the extracts with regard to the antibacterial activity of streptomycin against *K. pneumoniae*.

The synergistic effect of the extract and the drug and the variation in antibacterial activity induced by the different streptomycin/extract mixtures were demonstrated by interaction index curves established for each tested bacteria (Figure 2).

The maximum antibacterial activity was obtained in mixtures containing 25% of streptomycin. There were differences in interaction index (II) between the extracts when used against *S. aureus* and *B. cereus* (Figures 2a and 2b). The best II values were observed for EHE when used against *B. cereus,* which was the most susceptible bacteria to the streptomycin/extract mixture.

#### Evaluation of cytotoxicity in mammalian cells

**Figure 3:**
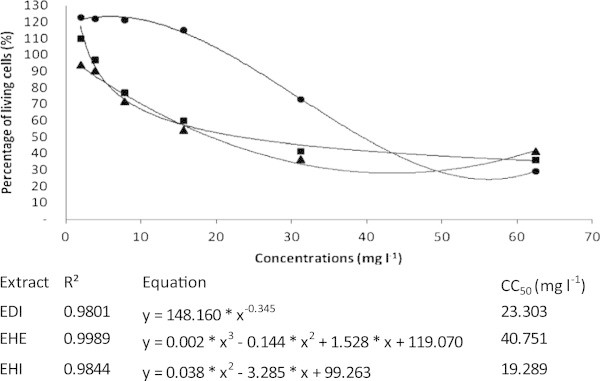
**Percentage of living MA104 mammalian cells as a function of the concentration of extracts from*Aristolochia cymbifera*determined by MTT colorimetric assay.** Three curves were obtained by non-linear regression to estimate the CC50 values (mg L^1^). For EHI (▲), EDI (■) and EHE (●), the CC50 values were 19.29, 23.30 and 40.75 mg L^-1^, respectively.

The results obtained in the *in vitro* cytotoxicity assays were plotted in terms of the percentage of living cells versus the concentration of the extract (Figure 3). A non-linear regression was generated to obtain an equation that demonstrates the relationship between the two variables. Adequate correlation coefficients (R^2^ of approximately 1.0) were obtained, indicating the efficiency of adopted models to estimate the CC50 (Figure 3). The hexanic extract was the least toxic with CC50 = 40.75 mg L^-1^.

## Discussion

The MIC results were significant with the exception of EHE against *S. aureus*, mainly because of the antibacterial activity against *S. aureus* and *K. pneumoniae*, which have shown resistance to several antibacterial drugs (Al-Masaudil *et al.*^1991^; Mangeney *et al.*^2000^). The low MIC values of the extracts against *B. cereus* indicate that the three extracts should be further studied to find active compounds to design new drugs. There were differences in antibacterial activity against *S. aureus*, *S. flexneri* and *K. pneumoniae* based on both extract polarity and bacterial strain. This fact can be associated with physiological differences between the microorganisms and the diversity and amounts of compounds in each extract.

The EHE and EDI extracts contain compounds with similar absorption bands in the UV region, but with different intensities. In the spectra of EHI and EDI, an absorption band at 400 nm was observed that was not found in the EHE spectrum (Figure 1). These data indicated that the use of solvents with different polarities allowed the extraction of distinct constituents in different concentrations. Some compounds have been isolated from *Aristolochia cymbifera* extracts, including 2-oxopopulifolic acid (Machado *et al.*^2005^), copalic acid, fargesin, hinokinin, kusunokinin, sesamin, epieudesmin, benznidazole (Sartorelli *et al.*^2010^), (5R,8R,9S,10R)-ent-3-cleroden-15-oic acid (Leitão *et al.*^1992^), (5R,8R,9S,10S)-ent-labd-14-en-8ß-ol (Lopes *et al.*^1988^), and (−)-kolavelool (Lopes *et al.*^1987^).

The highest concentrations of EDI, EHE and EHI in the streptomycin/extract mixture that showed a synergistic effect were 10.42 ± 3.61 mg L^-1^ (50% EDI), 6.25 ± 2.71 mg L^-1^ (75% EHE) and 6.25 ± 2.71 mg L^-1^ (75% EHI) against *K. pneumonia* (Additional file 1). Using the non-linear regression equations obtained from the cytotoxicity assay (Figure 3), the percentage of living cells at the above extract concentrations would be 68.00 ± 11.93, 122.54 ± 0.29 and 80.49 ± 10.77 respectively, each of which resulted in more than 50% living cells.

The *in vitro* toxic effect of *Aristolochia cymbifera* extracts on MA104 cells (Figure 3) is similar to that observed in studies that report high toxicity of the *Aristolochia* genus to human kidney cells, which was associated with aristolochic acids (Chen *et al.*^2010^). However, the checkerboard dilution test used in this study showed that lower extract concentrations improve the antibacterial activity of the mixture (see Additional file 1).

The 75% EHE mixture was generally the most effective solution against the studied bacteria (Additional file 1), containing a streptomycin concentration near the MIC of the antibiotics recommended for the treatment of *S. aureus* (oxacillin 0.5 mg L^-1^) (Auwaerter *et al.*^2013^; European Committee on Antimicrobial Susceptibility Testing 2013) and *B. cereus* (ciprofloxacin 0.25 mg L^-1^) (Turnbull *et al.*^2004^). Based on these data and *in vivo* cytotoxicity tests, which must be performed, the mixture of streptomycin plus the hexanic extract from *Aristolochia cymbifera* has good potential to be used as an alternative treatment against the studied bacteria. Even if the toxic effects of *A. cymbifera* are related to the aristolochic acids, this damage is associated with high cumulative doses of these compounds (Nortier *et al.*^2000^; Martinez *et al.*^2002^), which are unlikely to occur with the low extract concentration in the streptomycin/extract mixture that demonstrated antibacterial activity in this study. Additionally, the use of *A. cymbifera* extracts in addition to antibiotic drugs reduces the dose of streptomycin required for treatment, which can decrease the resistance of bacteria to the antibiotic and reduce the costs of antibiotic therapy.

Streptomycin is an aminoglycoside antibiotic that acts on the 30S subunit of the bacterial ribosome and prevents protein synthesis, causing bacterial death (Luzzatto *et al.*^1968^). Thus, one possible hypothesis for the synergic effect between streptomycin and *A. cymbifera* extracts may be that compounds that are present in the extracts increase membrane fluidity or permeability, helping the streptomycin molecules cross the bacterial membrane to enter the cytoplasm. Some compounds identified in *A. cymbifera*, such as copalic acid, have been shown to be able to disrupt the cell membrane (Souza *et al.*^2011^).

## Conclusion

The popular use of medicinal plants can sometimes be a good indicator of a new product source, as plants have several substances with different proprieties that can cause a specific biological effect. In this study, the antibacterial activity of ethanolic, hexanic and dichloromethane extracts form *A. cymbifera* stems was studied in addition to the antibacterial activity of streptomycin/extract mixtures. The cytotoxicity of the extracts on mammalian cells was also evaluated.

The results show that *A. cymbifera* extracts have compounds with potential as antibacterial drugs against *S. aureus*, *K. pneumoniae*, *B. cereus* and *S. flexneri*. The antibiotic/extract proportion affected the antibacterial activity of the mixture and the proportion that optimized the streptomycin's antibacterial activity contained 75 percent extract. At this percentage, the streptomycin’s activity was increased four-fold.

Although the three extracts were toxic to the mammalian cells, at the concentrations used in the 75 percent extract they were less toxic. Thus, to demonstrate the safety of the use of a mixture of streptomycin/*A. cymbifera* extracts and the mode of action of these extracts when combined with streptomycin, *in vitro* and *in vivo* toxicity assays should be performed in addition to mechanistic studies.

## Methods

### Plant extract preparation

Stem samples from *A. cymbifera* (Mart. & Zucc) were purchased from Nutri Comércio de Ervas Ltda. Company (São Paulo, Brazil) (Lot: MIH0111DM). Extracts were obtained by maceration using 50 g of the stems in ethanol, dichloromethane or hexane for three weeks (5 × 250 mL of each solvent). The mixtures were filtered and the solvents were removed under reduced pressure in a rotary evaporator at 45°C. The crude extracts (EDI= 1.15 g, EHE= 0.27 g and EHI= 3.38 g) were dissolved in DMSO and stored at −20°C. The extracts were analyzed to determine their absorption spectrum over a range of wavelengths between 200 and 900 nm.

### Microorganisms

Reference bacterial strains were chosen in accordance with their ability to cause enteric disorders or association with gastrointestinal disorders or food poisoning episodes as follows: *Staphylococcus aureus* (ATCC 29213), *Bacillus cereus* (ATCC 11778), *Klebsiella pneumoniae* (ATCC 4352) and *Shigella flexneri* (ATCC 12022). The bacteria were reactivated on nutrient agar plates.

### Determination of Minimum Inhibitory Concentration (MIC)

The MIC was determined using a microdilution assay in a 96-well microplate, as suggested by *National Committee for Clinical Laboratory Standards* (NCCLS 2003), with modifications. The inoculum was adjusted to 0.5 on McFarland’s scale and diluted to 10^6^ CFU mL^-1^ in nutrient broth (HiMedia Laboratories®, India), which was used as the growth medium. The initial concentration of the extracts was 500 mg L^-1^, which was decreased 50 percent for each subsequent microplate’s column. Streptomycin (Estreptomax®, Eurofarma Laboratories, São Paulo, Brazil) and dimethyl sulfoxide (DMSO) were the positive and negative controls. The plates were incubated for 24 h at 37°C and then 10 μL of 3-(4,5-dimethylthiazol-2-yl)-2,5-diphenyltetrazolium bromide (MTT) (Sigma®) was added to each well. A blue color indicated that there were living cells (active metabolism), and a yellow color indicated the absence of living cells.

### Checkerboard dilution test

The activity of *A. cymbifera* extracts supplemented with streptomycin against the four studied bacteria was assessed by the checkerboard dilution test, as described by Lee *et al.* (2012 and Kumar *et al.* (2012), with adaptations. Sequential dilutions of different proportions of the extract/streptomycin mixture were performed (with initial concentration of 50 mg L^-1^ in the first microplate’s column) using nutrient broth (HiMedia Laboratories®, India) as the growth medium. After 24 h of incubation at 37°C, the plates were read on a spectrophotometer (EMax Endpoint®), and the MIC was considered as the minimal concentration that demonstrated less than or equal to 10 percent of the control optical density or did not show any observable growth. The fractional inhibitory concentration index (FICI) was calculated using the equation:

with:

and

Values of FICI less than or equal to 0.50 were considered to be indicative of a synergic effect. Values ranging from 0.51 to 1.00 indicated an additive effect, values from 1.01 to 2.00 were considered as indifferent and values above 2.00 indicated an antagonist effect.The interaction index (II) was used to describe the degree to which the extract induced an increase in the activity of streptomycin and to compare the different effects between the extracts. The II was determined using the formula:

(See the derivation of the equation in Additional file 2).

### Assessment of cytotoxicity in mammalian cells

MA104 cells (ATCC) (rhesus monkey kidney cells) were cultured in Dulbecco's Modified Eagle Medium (DMEM) (Sigma®) supplemented with 6 percent of Fetal Bovine Serum (FBS) at 37°C in a 5 percent CO_2_ atmosphere in 96-well microplate until 95 percent confluence was reached. The wells were washed twice with phosphate-buffered saline (PBS) and 200 μL of extracts from 250 to 1.95 mg L^-1^ in DMEM with 2 percent of FBS were added. After 48 h of incubation, the medium was discarded and 20 μL of MTT was added to each well. After 1 h, 130 μL of DMSO was added, and the UV/Vis absorbance was measured with a spectrophotometer to determine the concentration of the extract that killed 50 percent of cells (CC50) Twentyman and Luscombe, 1987.

### Statistical analysis

All assays were performed in triplicate in three independent experiments. The mean ± SD were used to describe the results. To analyze the data, descriptive statistics, analysis of variance (ANOVA) and non-linear regression were applied with 95 percent confidence intervals Skoog, 2007.

## Electronic supplementary material

Additional file 1: **Effect of the interaction between streptomycin and ethanolic (EHE), dichloromethanic (EDI) and hexanic (EHE) extracts from*A. cymbifera.*** This file shows a detailed table of the MIC of each mixture of streptomycin/extract and its respective FICs and FICI. (DOCX 23 KB)

Additional file 2: **Derivation of the interaction index (II) equation.** This file shows the idea that led the authors to use a different method to calculate and define the interaction index (II). It also describes the II equation derivation in an easy to understand format (Straetemans *et al.*^2005^; Tallarida 2002). (PDF 173 KB)
