# Supplementary material for: Combination of extracts from Aristolochia cymbifera with streptomycin as a potential antibacterial drug
Source: Springerplus. 2013 Sep 3;2:430. doi: 10.1186/2193-1801-2-430 (PMC3771021; doi:10.1186/2193-1801-2-430)
Supplement: Supplementary file 1 — Additional file 1: Effect of the interaction between streptomycin and ethanolic (EHE), dichloromethanic (EDI) and hexanic (EHE) extracts fromA. cymbifera. This file shows a detailed table of the MIC of each mixture of streptomycin/extract and its respective FICs and FICI. (DOCX 23 KB) [file 40064_2013_499_MOESM1_ESM.docx]

**Additional File 1**

Table 2- Effect of the interaction between streptomycin and ethanolic (EHE), dichloromethanic (EDI) and hexanic (EHE) extracts from *A. cymbifera*

| \| Bacteria /  Substance (%) \| Drug MIC* \| \| FICs \| \| FICI \| Outcome \| \| --- \| --- \| --- \| --- \| --- \| --- \| --- \| \| Extract \| Streptomycin \| Extract \| Streptomycin \| \| *S. aureus* ATCC 29213 \|  \|  \|  \|  \|  \|  \| \| Drug alone \|  \| 5.00 ± 3.54 \|  \|  \|  \|  \| \| EDI 25 \| 2.60 ± 0.90 \| 7.81 ± 2.71 \| 0.01 \| 1.56 \| 1.57 \| I \| \| EDI 50 \| 4.17 ± 1.8 \| 4.17 ± 1.80 \| 0.02 \| 0.83 \| 0.85 \| A \| \| EDI 75 \| 3.91 ± 1.35 \| 1.30 ± 0.45 \| 0.02 \| 0.26 \| 0.28 \| S \| \| EHE 25 \| 2.6 ± 0.9 \| 7.81 ± 2.17 \| - \| - \| - \|  \| \| EHE 50 \| 2.6 ± 0.9 \| 2.60 ± 0.90 \| - \| - \| - \|  \| \| EHE 75 \| 2.73 ± 1.79 \| 0.91 ± 0.60 \| - \| - \| - \|  \| \| EHI 25 \| 2.08 ± 0.9 \| 6.25 ± 2.71 \| 0.01 \| 1.25 \| 1.26 \| I \| \| EHI 50 \| 3.65 ± 2.39 \| 3.65 ± 2.39 \| 0.02 \| 0.73 \| 0.75 \| A \| \| EHI 75 \| 3.12 ± 1.35 \| 1.04 ± 0.45 \| 0.02 \| 0.21 \| 0.23 \| S \| \| *K. pneumoniae* ATCC 4352 \|  \|  \|  \|  \|  \|  \| \| Drug alone \|  \| 10.00 ± 0.00 \|  \|  \|  \|  \| \| EDI 25 \| 7.29 ± 4.77 \| 21.88 ± 14.32 \| 0.02 \| 2.19 \| 2.21 \| An \| \| EDI 50 \| 10.42 ± 3.61 \| 10.42 ± 3.61 \| 0.03 \| 0.48 \| 0.50 \| S \| \| EDI 75 \| 6.25 ± 2.71 \| 2.08 ± 0.90 \| 0.01 \| 0.21 \| 0.22 \| S \| \| EHE 25 \| 8.85 ± 6.32 \| 26.56 ± 18.94 \| 0.04 \| 2.66 \| 2.69 \| An \| \| EHE 50 \| 12.50 ± 0.00 \| 12.50 ± 0.00 \| 0.05 \| 1.25 \| 1.30 \| I \| \| EHE 75 \| 6.25 ± 2.71 \| 2.08 ± 0.90 \| 0.02 \| 0.21 \| 0.23 \| S \| \| EHI 25 \| 6.25 ± 0.00 \| 18.75 ± 0.00 \| 0.01 \| 1.88 \| 1.89 \| I \| \| EHI 50 \| 8.33 ± 3.61 \| 8.33 ± 3.61 \| 0.02 \| 0.83 \| 0.85 \| A \| \| EHI 75 \| 6.25 ± 2.71 \| 2.08 ± 0.90 \| 0.01 \| 0.21 \| 0.22 \| S \| \| *B. cereus* ATCC 11778 \|  \|  \|  \|  \|  \|  \| \| Drug alone \|  \| 2.92 ± 1.56 \|  \|  \|  \|  \| \| EDI 25 \| 0.91 ± 0.60 \| 2.73 ± 1.79 \| 0.01 \| 0.94 \| 0.95 \| A \| \| EDI 50 \| 1.04 ± 0.45 \| 1.04 ± 0.45 \| 0.01 \| 0.36 \| 0.37 \| S \| \| EDI 75 \| 1.56 ± 0.68 \| 0.78 ± 0.39 \| 0.02 \| 0.27 \| 0.29 \| S \| \| EHE 25 \| 1.04 ± 0.45 \| 3.12 ± 1.35 \| 0.02 \| 1.07 \| 1.09 \| I \| \| EHE 50 \| 0.91 ± 0.60 \| 0.91 ± 0.60 \| 0.01 \| 0.31 \| 0.33 \| S \| \| EHE 75 \| 0.98 ± 0.34 \| 0.33 ± 0.11 \| 0.02 \| 0.11 \| 0.13 \| S \| \| EHI 25 \| 0.78 ± 0.00 \| 2.34 ± 0.00 \| 0.01 \| 0.80 \| 0.82 \| A \| \| EHI 50 \| 1.82 ± 1.19 \| 1.82 ± 1.19 \| 0.03 \| 0.62 \| 0.65 \| A \| \| EHI 75 \| 1.37 ± 0.90 \| 0.46 ± 0.30 \| 0.02 \| 0.16 \| 0.18 \| S \| \| *S. flexneri* ATCC 12022 \|  \|  \|  \|  \|  \|  \| \| Drug Alone \|  \| 4.58 ± 3.86 \|  \|  \|  \|  \| \| EDI 25 \| 1.56 ± 0.00 \| 4.69 ± 0.00 \| 0.01 \| 0.85 \| 0.86 \| A \| \| EDI 50 \| 3.13 ± 0.00 \| 3.13 ± 0.00 \| 0.02 \| 0.68 \| 0.70 \| A \| \| EDI 75 \| 3.12 ± 1.35 \| 1.04 ± 0.45 \| 0.02 \| 0.23 \| 0.25 \| S \| \| EHE 25 \| 2.08 ± 0.90 \| 6.25 ± 2.71 \| 0.01 \| 1.36 \| 1.37 \| I \| \| EHE 50 \| 1.56 ± 0.00 \| 1.56 ± 0.00 \| 0.00 \| 0.34 \| 0.34 \| S \| \| EHE 75 \| 2.73 ± 1.79 \| 0.91 ± 0.60 \| 0.01 \| 0.20 \| 0.21 \| S \| \| EHI 25 \| 1.30 ± 0.45 \| 3.91 ± 1.35 \| 0.01 \| 0.85 \| 0.86 \| A \| \| EHI 50 \| 2.08 ± 0.90 \| 2.08 ± 0.90 \| 0.01 \| 0.45 \| 0.46 \| S \| \| EHI 75 \| 3.12 ± 1.35 \| 1.04 ± 0.45 \| 0.02 \| 0.23 \| 0.24 \| S \| |  | |  | |  |  |
| --- | --- | --- | --- | --- | --- | --- | --- | --- | --- | --- | --- | --- | --- | --- | --- | --- | --- | --- | --- | --- | --- | --- | --- | --- | --- | --- | --- | --- | --- | --- | --- | --- | --- | --- | --- | --- | --- | --- | --- | --- | --- | --- | --- | --- | --- | --- | --- | --- | --- | --- | --- | --- | --- | --- | --- | --- | --- | --- | --- | --- | --- | --- | --- | --- | --- | --- | --- | --- | --- | --- | --- | --- | --- | --- | --- | --- | --- | --- | --- | --- | --- | --- | --- | --- | --- | --- | --- | --- | --- | --- | --- | --- | --- | --- | --- | --- | --- | --- | --- | --- | --- | --- | --- | --- | --- | --- | --- | --- | --- | --- | --- | --- | --- | --- | --- | --- | --- | --- | --- | --- | --- | --- | --- | --- | --- | --- | --- | --- | --- | --- | --- | --- | --- | --- | --- | --- | --- | --- | --- | --- | --- | --- | --- | --- | --- | --- | --- | --- | --- | --- | --- | --- | --- | --- | --- | --- | --- | --- | --- | --- | --- | --- | --- | --- | --- | --- | --- | --- | --- | --- | --- | --- | --- | --- | --- | --- | --- | --- | --- | --- | --- | --- | --- | --- | --- | --- | --- | --- | --- | --- | --- | --- | --- | --- | --- | --- | --- | --- | --- | --- | --- | --- | --- | --- | --- | --- | --- | --- | --- | --- | --- | --- | --- | --- | --- | --- | --- | --- | --- | --- | --- | --- | --- | --- | --- | --- | --- | --- | --- | --- | --- | --- | --- | --- | --- | --- | --- | --- | --- | --- | --- | --- | --- | --- | --- | --- | --- | --- | --- | --- | --- | --- | --- | --- | --- | --- | --- | --- | --- | --- | --- | --- | --- | --- | --- | --- | --- | --- | --- | --- | --- | --- | --- | --- | --- | --- | --- | --- | --- | --- | --- | --- | --- | --- | --- | --- | --- | --- | --- | --- | --- | --- | --- | --- | --- | --- | --- | --- | --- | --- | --- | --- | --- | --- | --- | --- | --- | --- | --- | --- | --- | --- | --- | --- | --- | --- | --- | --- | --- | --- | --- | --- | --- | --- | --- |
| * MIC values = mg l^-1^; I = Indifferent; A = Additive; S = Synergistic; and An = Antagonist effect. |  |  |  |  |  |  |
