# Supplementary material for: Combination of extracts from Aristolochia cymbifera with streptomycin as a potential antibacterial drug
Source: Springerplus. 2013 Sep 3;2:430. doi: 10.1186/2193-1801-2-430 (PMC3771021; doi:10.1186/2193-1801-2-430)
Supplement: Supplementary file 2 — Additional file 2: Derivation of the interaction index (II) equation. This file shows the idea that led the authors to use a different method to calculate and define the interaction index (II). It also describes the II equation derivation in an easy to understand format (Straetemans et al.2005; Tallarida 2002). (PDF 173 KB) [file 40064_2013_499_MOESM2_ESM.pdf]

Additional file 2

A simple method for describe and identify how much the extracts increased the antibacterial activity of the streptomycin was developed. Then, the focus in this deduction is analyze the effect of the mixture on the streptomycin activity. Some models for interaction index were described (*33, 34*). There are many similarities between them and the fractional inhibitory concentration index (FICI). However, some of them are more complex and focus in both drugs, giving a general interaction index.

We know that the MIC is reversely proportional at antimicrobial activity (AA) (the smaller MIC value, the bigger activity):

$AA \propto 1/MIC$

Then, adding a proportionality constant α (where $\alpha \neq 0$), we can write:

$AA = \alpha.(1/MIC)$

AA is function of only MIC value, and MIC depends of other parameters (inoculum concentration, pH, action mechanisms of the drug and the extract).

An interaction factor (IF) can be defined as a fraction of the activity when the AA of the mixture streptomycin + extract ($AA_{streptomycin + extract}$) is divided to AA of the alone streptomycin ($AA_{streptomycin}$):

$IF = (AA_{streptomycin + extract})/(AA_{streptomycin})$

The interaction index (II) may show numerically how much the activity of streptomycin was increased and can be described by;

$II = IF - 1$, where 1 represent the total streptomycin activity $[(AA_{streptomycin})/(AA_{streptomycin}) = 1]$.

If so, when the II is lower than zero, the interaction between the both drugs is negative. When the II is zero, there is not interaction between the both. An II largest than zero indicate a positive effect in the interaction between the both, and the bigger II value, the bigger it is the capacity of the extract of increase the antimicrobial activity of streptomycin.

As $IF = (AA_{streptomycin + extract})/(AA_{streptomycin})$ and $AA = \alpha.(1/MIC)$;

$IF = [\alpha.(1/MIC_{streptomycin + extract})]/[\alpha.(1/MIC_{streptomycin})] =$

$= MIC_{streptomycin}/ MIC_{streptomycin + extract} = 1/ FIC_{streptomycin}$

Then, the interaction index is defined by:

$II = (1/FIC_{streptomycin}) -1$

This method is interesting because: i) it is very simple to calculate; ii) it allows create a graphic of II versus percentage of drug at mixture, what ease the data interpretation (negative, neutral and positive interaction), the activity optimization analysis and the comparison between the different extract's characteristics and effect on bacteria.
